# MicroRNAs contribute to postnatal development of laminar differences and neuronal subtypes in the rat medial entorhinal cortex

**DOI:** 10.1007/s00429-017-1389-z

**Published:** 2017-03-04

**Authors:** Lene C. Olsen, Kally C. O’Reilly, Nina B. Liabakk, Menno P. Witter, Pål Sætrom

**Affiliations:** 1 Department of Cancer Research and Molecular Medicine, Norwegian University of Science and Technology, Trondheim, Norway; 2 Kavli Institute for Systems Neuroscience and Centre for Neural Computation, Norwegian University for Science and Technology, Trondheim, Norway; 3 Department of Computer and Information Science, Norwegian University for Science and Technology, Trondheim, Norway; 4 Bioinformatics core facility-BioCore, Norwegian University of Science and Technology, Trondheim, Norway

**Keywords:** Medial entorhinal cortex, MiRNA, Brain development, Stellate neurons

## Abstract

The medial entorhinal cortex (MEC) is important in spatial navigation and memory formation and its layers have distinct neuronal subtypes, connectivity, spatial properties, and disease susceptibility. As little is known about the molecular basis for the development of these laminar differences, we analyzed microRNA (miRNA) and messenger RNA (mRNA) expression differences between rat MEC layer II and layers III–VI during postnatal development. We identified layer and age-specific regulation of gene expression by miRNAs, which included processes related to neuron specialization and locomotor behavior. Further analyses by retrograde labeling and expression profiling of layer II stellate neurons and in situ hybridization revealed that the miRNA most up-regulated in layer II, miR-143, was enriched in stellate neurons, whereas the miRNA most up-regulated in deep layers, miR-219-5p, was expressed in ependymal cells, oligodendrocytes and glia. Bioinformatics analyses of predicted mRNA targets with negatively correlated expression patterns to miR-143 found that miR-143 likely regulates the Lmo4 gene, which is known to influence hippocampal-based spatial learning.

**Electronic supplementary material:**

The online version of this article (doi:10.1007/s00429-017-1389-z) contains supplementary material, which is available to authorized users.

## Introduction

The entorhinal cortex (EC) is implicated in the formation of memory. In particular, the medial part of the entorhinal cortex (MEC) is important for spatial memory and navigation (Derdikman and Moser 2010; Eichenbaum et al. 2012). The MEC has a laminar structure in which each layer has dominant cell types, differing in physiological properties and connectivity (Canto and Witter 2012; Greenhill et al. 2014). The laminar topography is also evident with regards to gene expression (Ramsden et al. 2015) and in certain pathological conditions; neuronal death and neurofibrillary tangles form in layer II (LII) at early stages of Alzheimer’s disease (Gomez-Isla et al. 1996), and LII also displays abnormalities in schizophrenia (Arnold 2000), whereas layer III of MEC has been implicated in temporal lobe epilepsy (Schwarcz et al. 2000). Origins of laminar differences in MEC are therefore important for both normal and pathological MEC functions.

The overall structure of MEC is observable at birth, but there is substantial postnatal development of MEC cells, physiological properties, and projections, continuing at least until postnatal day 28 (P28) (Burton et al. 2008). With respect to cell types, it is established that the adult rat MEC contains several types of spatially tuned neurons, including grid, border, and head direction cells, as well as speed modulated neurons and conjunctive cells that display both grid and head direction properties (Rowland et al. 2016). Spatially tuned neurons are unevenly distributed across MEC layers, with the majority of grid cells found in LII (Sargolini et al. 2006). The dominant cell type (67%) in LII is the glutamatergic stellate neuron (Gatome et al. 2010), thereby making it likely that this neuron corresponds to the grid cell, although this has been debated by several groups (Domnisoru et al. 2013; Moser and Moser 2013; Tang et al. 2014). General postnatal development in all cortical areas of the rat includes glial cell production and specialization, myelination, and an overproduction of synapses in infancy followed by pruning in juveniles (Downes and Mullins 2014; Semple et al. 2013). Extensive synaptogenesis and dendrite formation also occurs in the MEC, and the stellate neurons double their spine density between P14 and P18 (Burton et al. 2008). The physiological properties of MEC neurons also mature during the first postnatal weeks, with stellate cells exhibiting falling resistance and increasing resonance (Burton et al. 2008; Langston et al. 2010). Whereas grid-like cells are present soon after the eyes open (~P14), the grid cell properties stabilize around 4 weeks of age (Langston et al. 2010). Other spatially tuned cells mature earlier. Head direction cells appear adult-like upon eye opening, and boundary cells display adult-like firing when the rats begin to explore their environment (Bjerknes et al. 2014; Langston et al. 2010). The major projection from MEC to hippocampus already shows adult-like topography within the first postnatal week (Deng et al. 2007; O’Reilly et al. 2015). However, this MEC-to-hippocampus projection density is not considered adult-like until P10 (Deng et al. 2007). Changes through the first few weeks after birth are therefore fundamental for the properties of the adult MEC.

As changes in spatio-temporal gene expression underlie general postnatal development, layer-specific gene expression likely guides the cellular, physiological, and structural changes occurring postnatally in each MEC layer. Reelin (Reln, see Supplementary Table 1 for full gene names) plays a role in the development of neuron morphology and layer-specific connections in both the EC and the rest of the cortex (Borrell et al. 1999; Stranahan et al. 2013), but little is known about other molecular changes orchestrating laminar specialization.

MicroRNAs (miRNAs) are small non-coding RNA molecules that regulate gene expression after transcription and are important in many aspects of central nervous system development (Olde Loohuis et al. 2012). Many miRNAs are differentially expressed in various brain regions (Olsen et al. 2009), reflecting the brain region specific regulation of messenger RNAs (mRNAs). Some miRNAs also regulate mRNAs locally at the synapse and play a role in the development of neuronal morphology and regulation of synaptic plasticity (Olde Loohuis et al. 2012), whereas other miRNAs are involved in specifying neuronal subtypes (Stappert et al. 2015).

**Fig. 1:**
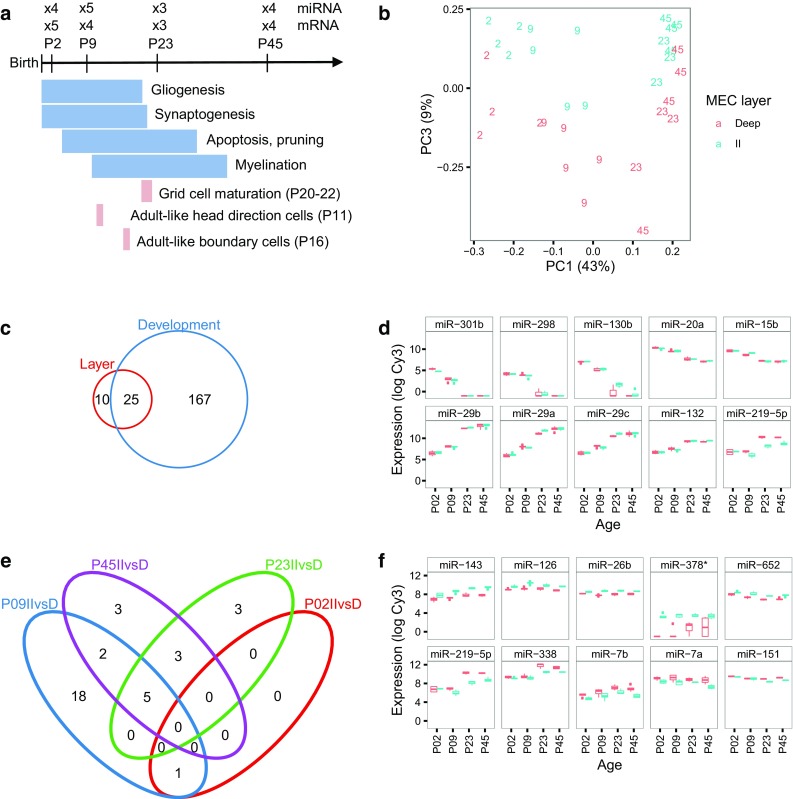
Analysis of miRNA expression in MEC. **a** Overview of the laminar gene expression experiment. Time points and number of biological replicates used for miRNA and mRNA expression analysis in relation to the timing of major maturation events (*blue*) and the known maturation time points of navigational cell types in MEC (*tan*). **b** Principal component analysis of the miRNA expression samples. The *x* and *y axes* show the first and third principle components (PC1 and PC3); the axes text specify the percentage of expression variation explained by the respective PCs. PC2 depicted a mixture between age and layers, whereas PC3 clearly reflected laminar differences. Each *character string* represents the age of the animal and an age-specific number identifying LII (*turquoise*) and LDeep (*red*) samples from the same animal. **c** *Venn diagram* showing the number of differentially expressed miRNAs between layers (*red*) and between ages (*blue*, P2/P9 vs. P23/P45, LFC = 0, BH < 0.05), and their overlap. **d** Expression of the five most significant down-regulated (*top*) and up-regulated (*bottom*) miRNAs between ages. **e** *Venn diagram* showing the number of miRNAs DE between layers at P2 (*red*), P9 (*blue*), P23 (*green*), and P45 (*purple*), and their overlap. **f** Expression patterns of the five most significant miRNAs up-regulated in LII (*top*) and up-regulated in LDeep (*bottom*) across development

Knowing the importance of miRNAs in orchestrating neuronal development and most other cellular processes, we hypothesized that miRNAs contribute to the laminar and neuronal subtype specialization within the MEC in general, and the stellate cells in particular. We therefore measured miRNA expression in LII, where the stellate cells are abundant, and the deeper layers (layers III–VI, LDeep) of the MEC of rats during postnatal development. We sampled at P2, P9, P23, and P45, as these ages represent early, intermediate, late, and completed postnatal developmental time points, respectively, and thereby cover major developmental events, including maturation of grid cells and onset of hippocampal spatial learning (Fig. 1a). In addition, we examined the miRNA profile of the stellate cells compared to the rest of the MEC at an early postnatal age (P4/5). We found several miRNAs to be differentially expressed between layers and cell types (stellate vs. non-stellate cells). To identify more likely target gene candidates for interesting miRNAs in the MEC, we measured ribosomal RNA-depleted total RNA gene expression in LII and LDeep at the same time points. MicroRNAs with increased expression level in older rats compared to younger rats appear to play a role in the cell cycle and early developmental events such as axon guidance, whereas miRNAs with opposite expression patterns seem to have important roles in synaptic transmission, plasticity and myelination. Important for navigation, miRNAs with decreased expression in older rats also appear to regulate locomotor behavior. Two miRNAs, miR-143 and miR-150, were up-regulated both in LII and in stellate neurons. The most significant up- and down-regulated miRNAs in LII (miR-143 and miR-219-5p, respectively) were validated by in situ hybridization. By analyzing for enriched ontology terms for their predicted, negatively correlated target genes, we found that miR-219-5p appears to regulate myelination, while miR-143 likely contributes to the specification of neuronal subtypes.

## Methods

### Animals

Long Evans pups were used for the studies presented here. Breeding harems consisted of one male rat and up to three female rats. The harems were housed in an enriched environment with toys and access to food and water ad libitum. The rats were maintained on a 12 h reversed light/dark schedule. Cages were examined morning and evening, and the day pups were observed was considered P0. Litters were culled to approximately ten pups by P3. Pups were allowed to remain with the mother in the nest until weaning at P21. All procedures were approved by a local ethics committee according to Norwegian and EU regulations.

### Collection and dissection of laminar tissue samples

Rats aged P2, P9, P23, and P45 were anesthetized with isoflurane and decapitated. The brains were quickly harvested and kept in ice cold artificial spinal fluid. Horizontal 400 µm sections were cut on a Leica VT 1000 S microtome and put in RNA*later* ^®^ (AM7020, Ambion, Austin, TX, USA). The tissue sections were kept at 4 °C until dissection. Bilateral dissection of layer II and layers III-VI (LDeep) of MEC was performed, while watching the tissue through a dissection microscope (Zeiss Discovery V8 stereomicroscope) applying architectonic criteria (Boccara et al. 2015; O’Reilly et al. 2015; Paxinos and Watson 2007) to unstained tissue. In horizontal sections, MEC is easily recognized using transmitted and reflected white light by the marked shape of the cortex, the prominent white, opaque lamina dissecans and the radial organization of the layers deep to the latter. Layer II neurons are large spherical neurons, which differ markedly in level of opacity from those in layer III. The medial border between MEC and parasubiculum is characterized by the loss of the differences between layers II and III, while the border with the laterally adjacent postrhinal cortex is characterized by the loss of the large spherical neurons in layer II. All dissections avoided border regions, i.e., were taken centered in the identified MEC and specific layers. The dissected MEC tissue was transferred into fresh RNA*later* ^®^ and kept at −20 °C until RNA purification.

### RNA purification and quality control

Total RNA was purified using the RNAqueous^®^-Micro kit (AM1931, Ambion, Austin, TX, USA). The manufacturer’s instructions were followed, except that wash steps 2 and 3 were modified to include rolling of the tubes and incubation with the wash buffer for 1 min, and the ethanol added for precipitation was increased to 1.25× lysis buffer volume to include small RNAs. The RNA was eluted in 2 × 10 µl RNAqueous Elution Buffer. In the layer samples for miRNA microarray analysis, four of the tissue samples were extracted using the mirVana^™^ kit (Ambion, Austin, TX, USA). The RNA was eluted in 2 × 50 µl mirVana Elution Buffer. We used the Norgen Total RNA purification kit (Norgen Biotek, Canada) to purify RNA from whole medial entorhinal tissue.

RNA yield was determined using the NanoDrop 1000 spectrophotometer or the Qubit^®^ 2.0 Fluorometer (Life Technologies, Carlsbad, CA), and the quality was assessed with the Agilent BioAnalyzer 2100 Nano chip. Only samples with a RIN value of 8.5 or above were included for further analysis. Isolated RNA samples were stored at −80 °C until further use.

### Microarray analysis

The total RNA from the laminar samples were shipped on dry ice to IMGM^®^ Laboratories in Martinsried, Germany, for microarray analysis. 100 ng total RNA per sample were introduced into the labeling reaction. Prior to this, the total RNA samples were spiked with in vitro synthesized oligonucleotides (MicroRNA Spike-In Kit, Agilent Technologies), which serve as an internal labeling control for linearity, sensitivity and accuracy. Microarray analyses were done on Rat miRNA Microarrays, Release 15.0 (Agilent Technologies, AMADID 029200, 8 × 15 K format), according to the manufacturer’s instructions. The miRNA expression data has been submitted to the Gene Expression Omnibus (GEO) database with accession number GSE85753.

### Retrograde labeling of stellate cells

On P2, Long Evans rats were anesthetized with isoflurane in an induction chamber and then moved to a stereotaxic frame. Rats were placed in a neonatal mask (Kopf, model 973-B, Tujunga, CA, USA), head fixed using zygoma ear cups (Kopf, model 921, Tujunga, CA, USA), while isoflurane anesthesia was maintained for the duration of the surgery. Saline was administered subcutaneously during the course of the surgery (up to 50 μl/g body weight). Rats were also administered 5 μg/g body weight of rimadyl as an analgesic. The retrograde tracer dioctadecyloxacarbocyanine (DiO, cat# D275, Invitrogen, Molecular Probes, Eugene, OR, USA) dissolved in ethanol:dimethyl sulfoxide (9:1 EtOH:DMSO) at a concentration of 3 mg/mL was iontophoretically injected into the dentate gyrus through glass micropipettes (outer diameter of ~30 μm). We used a 5- to 7-μA alternating positive current (6 s on/6 s off for 15 min) delivered by a digital current source (Stoelting Europe, Dublin, Ireland). After recovery under a heat lamp, rat pups were returned to maternal care for 24–48 h.

At P4/5, the animal was decapitated while under deep isoflurane anesthesia. Retrogradely labeled and non-labeled MEC was dissected from both hemispheres, minced into smaller pieces in a bath of ice cold Hibernate A (no phenol red, Brain Bits) containing 0.5 mM Glutamine and B27 supplement (17504-044, Invitrogen, Carlsbad, CA, USA) to support neuronal viability. The pieces were aspirated into a tube containing Hibernate A at 4 °C using a wide bore pipette.

P4/5 was chosen to ensure easier dissociation in the absence of myelin, and also to allow shorter transportation times for the dye.

### Cell dissociation

The dissected entorhinal tissue was dissociated using a protocol adapted from Brewer (Brewer 1997). Briefly, the tube containing the pieces of entorhinal cortex in Hibernate A was kept at 30 °C for 5 min with occasional resuspension of the pieces. The Hibernate A was then aspirated and replaced by 1 ml of pre-warmed Hibernate A with 2 mg/ml papain (LS003119, 26.1 U/mg, 79% protein, Worthington Biochemical Corporation, Lakewood, NJ, USA). The tube was incubated at 30 °C for 30 min with resuspension of pieces every 5 min, after which the enzyme solution was replaced with 0.5 ml Hibernate A/B27 at 30 °C. DNase (D4527-10KU, Sigma–Aldrich Co. LLC, St. Louis, MO, USA) was added to the suspension solution (0.3 U/ml), followed by a 5 min incubation at room temperature before gentle trituration with a Pasteur pipette. The suspension was allowed to settle for 2 min before the supernatant was transferred to a fresh tube and the pellet resuspended in 0.5 ml Hibernate/B27. This trituration procedure was repeated twice more using Pasteur pipettes with consecutively smaller openings.

The resulting cell suspension was centrifuged on an Optiprep gradient (1114542, Axis-Shield PoC, Oslo, Norway) according to the manufacturer’s application sheet C29. The top two ml and the densest layer of debris were removed, before diluting the resulting suspension 1:2 with Hibernate A/B27.

### Fluorescence activated cell sorting (FACS)

The retrogradely labeled cells were separated from the non-labeled cells using a FACS Diva cell sorter (BD Biosciences). Cells were first gated based on forward and side scatter using Calcein Blue AM fluorescence (final concentration 2 μM, C1429, Invitrogen, Carlsbad, CA, USA) on a small portion of the cell suspension to determine the viable cell population. Propidium iodide (final concentration 1 μg/ml, Invitrogen, Carlsbad, CA, USA) was used to exclude apoptotic/dead cells, and fluorescence in the green channel was used to select retrogradely labeled DiO positive cells. 10,000–100,000 cells were sorted directly into RNAqueous^®^-Micro lysis buffer and stored at −80 °C before RNA purification. Cells from both hemispheres were pooled.

### Taqman qPCR array analysis

The total RNA purified from the FACS sorted samples was shipped on dry ice to IMGM^®^ Laboratories in Martinsried, Germany, for TaqMan array analysis. The TaqMan^®^MicroRNA Reverse Transcription Kit (Applied Biosystems) in combination with the Megaplex^™^ RT Primers, Rodent Pool Set v3.0 for TaqMan^®^MicroRNA Assays (Applied Biosystems) was used in a multiplex reverse transcription of miRNA into single stranded cDNA. In total, two (A + B) separate RT reactions were carried out for each sample with >1 ng of total RNA per reaction according to the manufacturer’s instructions. 2.5 µl of each cDNA (A + B) were amplified using the TaqMan^®^ PreAmp Master Mix together with the Megaplex^™^ PreAmp Primers, Rodent Pool Set v3.0 according to manufacturer’s instructions. The software ViiA7 Software v1.2 (Applied Biosystems) was used for instrument control and raw data control. For each well, cycle threshold (Ct) values, i.e., the cycle number where the amplification curve clearly exceeds the background, were calculated in the software ViiA7 Software v1.2 using the default analysis settings. The TaqMan miRNA array data has been submitted to the GEO database with accession number GSE85752.

### Deep sequencing

The Illumina TruSeq^®^ Stranded Total RNA HT with Ribo-Zero Gold was used for library preparation according to the manufacturer’s recommendations, and the resulting libraries were sequenced on the Illumina HiSeq 2500 (Illumina, San Diego, CA) using 2 × 100 bp paired end sequencing by the Genomics Core Facility at NTNU, Trondheim, Norway. The use of multiplex adapters allowed all samples to be run across all lanes. The RNA-seq data has been submitted to the GEO database with accession number GSE85789.

The sample for small RNA sequencing was prepared according to Illumina’s small RNA TruSeq protocol, and sequenced using 50 bp single read on one lane on the Illumina HiSeq 2000 at the Norwegian High Throughput Sequencing Centre at Oslo University Hospital, Oslo, Norway. The small RNA-seq data has been submitted to the GEO database with accession number GSE85788.

### In situ hybridization

We perfused two rats aged P23 intracardially with Ringers solution followed by 4% paraformaldehyde/PBS. The brains were extracted and postfixed for 24 h in 4% paraformaldehyde followed by cryoprotection in 0.5 M sucrose/PBS solution for 48 h (both at 4 °C). The brains were snap frozen in TissueTek OCT (Sakura, Japan) by immersion in an isopentane/dry ice slurry. 14 µm sagittal sections were cut by cryostat (Microm HM 560, Thermo Scientific) and mounted on SuperFrost^®^ Plus slides (Thermo Scientific). The sections were dried for 45 min, and kept at −20 °C until further use.

In situ hybridization was performed with locked nucleic acid probes from Exiqon (Vedbaek, Denmark). The slides were removed from the freezer and allowed to thaw and dry at room temperature for 15 min, before incubation with 1.25 or 1.5 μg/ml Proteinase K for 10 min at 37 °C. The rest of the procedure was according to Exiqon’s miRCURY LNA^™^ microRNA ISH Optimization Kit (FFPE) Instruction manual v2, except that we used 40 µl of probe solution and covered with RNAseAWAY-treated parafilm. A Dako Hybridizer (Dako, Denmark) was used for all incubations. Imaging of sections was performed with a Zeiss Axio Imager M2 microscope, with a 5× magnification objective.

### Data analysis

Statistical and other analyses were performed in R, unless otherwise stated. The miRNA microarray results were analyzed using the AgiMicroRna package (Lopez-Romero 2011) with the filterMicroRna function and quantile normalization of the total gene signal calculated by the Agilent Feature Extraction software. Limma with empirical Bayes correction was used to ascertain differential expression (v.3.18.13) (Smyth 2004).

We used the HTqPCR package (Dvinge and Bertone 2009) to analyze the TaqMan array data, with deltaCt normalization, and the filterCtData function to filter out “Undetermined” and “Unreliable” results. Differential expression was determined using the limmaCtData function.

For the Illumina small RNA sequencing data, raw reads were processed using CASAVA (v. 1.8.2 Illumina), and the quality of the reads assessed by FastQC (v0.11.2, http://www.bioinformatics.babraham.ac.uk/projects/fastqc/). We used Cutadapt v.1.0 (Martin 2011) to remove all reads below 15 nt and adapter sequences, as well as trimming low quality ends (Phred < 20). We also removed all reads with an average Phred quality score below 20 using FastQ Quality Filter (Fastx tool kit v.0.0.13, http://hannonlab.cshl.edu/fastx_toolkit/). The resulting reads were aligned to the Rn4 genome with Bowtie v.0.12.7 (Langmead et al. 2009), allowing up to ten alignments per read. The mapped reads were annotated and counted using HTSeqCount v.0.5.4p3 (Anders et al. 2015) with annotation data from miRBase v. 20.

For the Illumina paired end RNA sequencing analysis, raw reads were processed using bcl2fastq (v.1.8.4, Illumina). We removed adapter sequences, reads below 20 nt, and low quality bases at the ends using Trimmomatic (v.0.33) (Bolger et al. 2014). The sequences were aligned to the rat reference genome (Rn6) using STAR [v2.4.0, (Dobin et al. 2013)]. Annotation and gene counts were obtained using featureCounts of the Subread package (v1.4.6-p1) (Liao et al. 2014), using RefSeq gene annotations for Rn6 downloaded from UCSC on April 8, 2015. The counts were transformed with the Limma voom function (Law et al. 2014), and normalized by TMM (Robinson and Oshlack 2010) and quantile normalization (Bolstad et al. 2003). We used Limma with empirical Bayes to identify differentially expressed genes (v.3.26.9) (Ritchie et al. 2015; Smyth 2004).

Overlaps between differentially expressed genes and miRNAs in different contrasts were visualized with the Vennerable package (https://github.com/js229/Vennerable), and overlaps between miRNA expression technologies were visualized with the Venneuler package (http://www.rforge.net/venneuler/). We grouped DE miRNAs into co-expression modules based on Pearson correlation, and groups were identified using the Partitioning Algorithm and the Recursive Thresholding (PART) method in the CRAN package clusterGenomics (Nilsen et al. 2013).

We downloaded validated targets for miRNAs of interest from miRTarBase (Hsu et al. 2014). Predicted, conserved targets for the same miRNAs were obtained from TargetScan v.6.2 (Grimson et al. 2007). We downloaded the “Conserved_Site_Context_Scores.txt” file, and selected rat genes with a context + score below −0.1. The most important target predictions were later examined against TargetScan v.7. MirbaseTracker (Van Peer et al. 2014) allowed miRNA naming conversions between different versions of miRBase.

To identify negatively correlated potential and validated targets of the differentially expressed miRNAs, we set three requirements. First, the mRNA had to be listed as a predicted, conserved target in TargetScan or as a validated target in miRTarBase. Second, both the miRNA and the mRNA had to be differentially expressed between LII and LDeep, or between younger (P2/P9) and older (P23/P45) animals. Third, the expression pattern of the miRNA had to be significantly negatively correlated with that of its mRNA target (Spearman’s rho <−0.5).

#### Gene ontology analysis

GO, KEGG pathway, and REACTOME pathway enrichment analyses for *Rattus norvegicus* genes were performed using the Bioconductor package gProfileR (Reimand et al. 2007). Only genes displaying a log fold change (LFC) of more than 0.5 were included for the analysis. We also performed this analysis on predicted, conserved target genes of differentially expressed miRNAs that were expressed in the MEC. The *p* values for the enriched terms were adjusted with the FDR method, and only terms with an adjusted *p* value below 0.05 were included. We calculated the odds ratio for each enriched term using the Fisher’s exact test. After removing terms containing more than 2500 genes, the results were curated manually to remove redundant and/or uninformative terms.

#### miRNA cluster analysis

We downloaded genomic coordinates of the different miRNAs from miRBase, and defined a miRNA cluster as a minimum of two miRNAs, where each miRNA was located within 10 kb of the next miRNA member of the cluster.

## Results

### miRNA expression in MEC layers during postnatal development

To identify miRNAs that are important for postnatal development of the entorhinal cortex in general, and for laminar development within the MEC in particular, we performed miRNA microarray analysis on total RNA from LII and LDeep of rats at four different postnatal ages (P2, P9, P23, and P45; Fig. 1a, Supplementary Fig. 1). Because stellate cells are enriched in LII, we hypothesized that the results for LII would include findings relevant for this neuronal subtype.

Principal component analyses of the miRNA expression data showed that the main variation in the data separated the early (P2) and intermediate (P9) ages from the late (P23/P45) ages (Fig. 1b, component 1). To identify miRNAs that showed robust expression differences during development, we grouped the younger (P2/P9) and older (P23/P45) animals. After filtering and normalization (see methods), we found 192 miRNAs to be differentially expressed between ages (Fig. 1c; Supplementary Table 2; Benjamini-Hochberg (BH) adjusted *p* value < 0.05), of which 88 were down-regulated in older compared to younger animals (with higher expression at P2/P9 compared to P23/P45 rats), and 104 were up-regulated in older compared to younger animals (higher expression at P23/P45 than at P2/P9). The members of the miR-29 family showed the highest increase in expression level from younger to older animals (Fig. 1d; log fold-change (LFC) of up to 5.5 from P2/P9 to P23/P45).

Fewer miRNAs displayed differential expression between LII and LDeep, with only one miRNA being differentially expressed at P2, 26 differentially expressed at P9, 11 at P23, and 13 at P45 (35 unique miRNAs, Fig. 1c, e, f, Supplementary Table 2). This was also evident from the principal component analysis, where only nine percent of the variability in the data was explained by laminar differences (PC3) (Fig. 1b). When all ages were combined, 44 miRNAs were differentially expressed between layers (Supplementary Table 2). Of these 44, 27 miRNAs showed higher expression in LII than in LDeep, and 17 showed higher expression in LDeep than in LII. Most miRNAs that showed expression differences between layers also displayed differential expression levels between ages.

In summary, these results point to radical changes in miRNA expression during postnatal development of the MEC and identify several miRNAs that have different expression patterns in MEC LII compared with deep layers.

### Functional analysis of laminar genes and predicted targets of laminar miRNAs

**Fig. 2:**
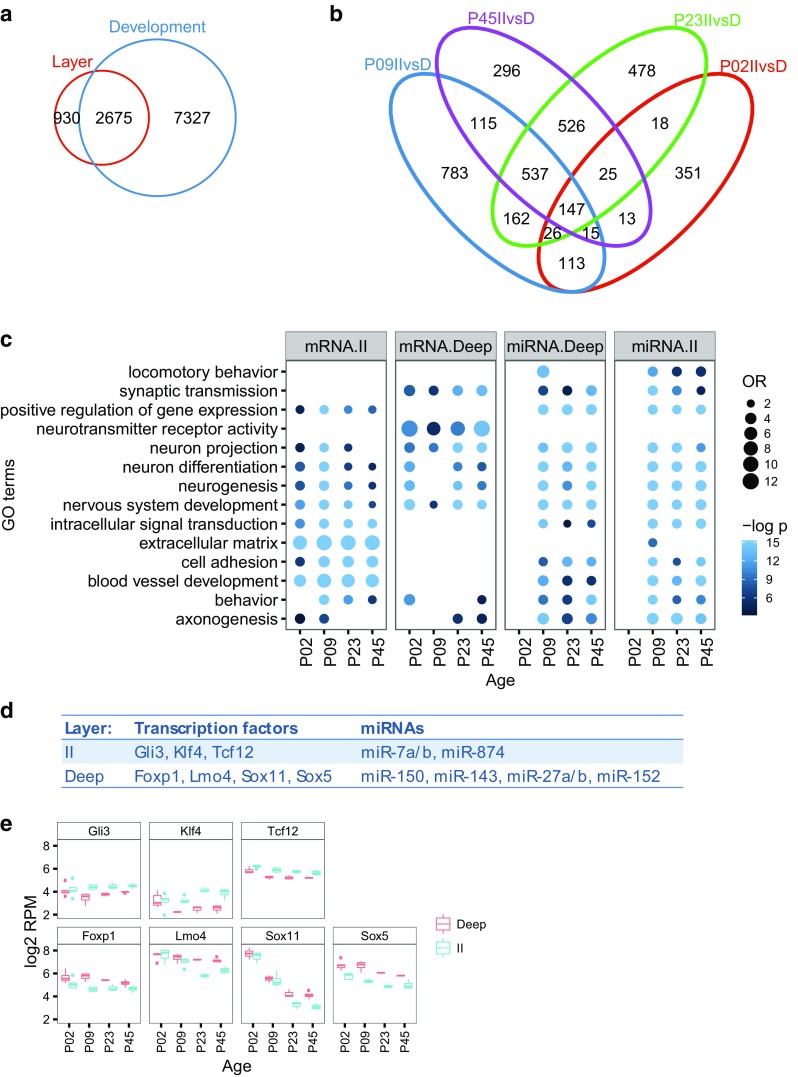
MEC mRNA expression and enrichment terms. **a** *Venn diagram* showing the number of differentially expressed mRNAs between layers (*red*) and between ages (*blue*, P2/P9 vs. P23/P45, LFC = 0, BH < 0.05), and their overlap. **b** *Venn diagram* showing the number of mRNAs differentially expressed between layers at P2 (*red*), P9 (*blue*), P23 (*green*), and P45 (*purple*), and their overlap. **c** Functional characterization of laminarly enriched mRNAs and targets of laminarly enriched miRNAs across development. We used gProfileR to search for enriched ontology terms for mRNAs up-regulated in LDeep (mRNA.Deep) and LII (mRNA.II), and for validated and predicted miRNA targets expressed in MEC for miRNAs up-regulated in LII (miRNA.II) and in LDeep (miRNA.Deep) for each age group. The color intensity reflects the statistical significance (negative log adjusted *p* value), and the size of the circles the odds ratio calculated by Fisher’s exact test. For illustration purposes, all OR and negative log *p* values above a maximum value of 12 and 15, respectively, were rounded down to these maximum values. **d** Transcription factors involved in neuron differentiation that are negatively correlated and predicted, conserved targets of miRNAs up-regulated in LII and up-regulated in LDeep. **e** Expression patterns of the transcription factors from d

As miRNA regulation primarily causes mRNA degradation (Guo et al. 2010), we expected negatively correlated expression patterns for many miRNAs and their target genes (Wang and Li 2009). To identify potential target genes of our differentially expressed miRNAs, we used ribosome-depleted total RNA sequencing to measure mRNA expression at the same time points and layers as for the miRNA expression analysis (Fig. 2a, b, Supplementary Table 3). This analysis also detected preliminary miRNA molecules (pre-mirs), and the expression of these correlated well with the corresponding mature miRNAs measured by microarrays, corroborating the findings from our microarray analyses (Supplementary Analyses SA1, Supplementary Fig. 2).

We used the gProfileR tool to identify common functions of the mRNAs differentially expressed between layers at each postnatal age tested (Fig. 2c). The same was done for predicted and validated targets of miRNAs differentially expressed at the different time points, except for P2. The only miRNA differentially expressed at P2 had neither predicted, nor validated targets in the databases we used for the analyses (see “Methods”).

Because morphology, connectivity, and physiological properties differ between the MEC layers, and because these properties develop in the time period tested, we expected to find GO terms and pathways involved in neural cell development, axon guidance, and ion channels. Indeed, mRNAs differentially expressed between layers were enriched in GO terms related to neuron projection and differentiation, irrespective of whether the mRNAs were up in LII or in LDeep. The mRNAs up-regulated in LII were particularly enriched in extracellular matrix proteins, cell adhesion, and angiogenesis, whereas genes up-regulated in LDeep were enriched in terms linked to neuron projection and synaptic activity. Many of the terms enriched for differentially expressed genes, such as the neuron development, angiogenesis, and adhesion terms, were also enriched for predicted or validated targets of laminar miRNAs. Strikingly, the targets for both up- and down-regulated miRNAs had many significant terms in common. This could reflect fine-tuning of gene expression between layers by miRNAs, which in turn could contribute to the laminar specialization.

We asked if the differentially expressed mRNAs included in the “neuron differentiation” category included miRNA-regulated transcription factors that potentially drive the differentiation or maintenance of the laminar neuronal subtypes. We used versions 6 and 7 of TargetScan to determine the context scores—a measure of a miRNA’s affinity to its predicted target site—of the differentially expressed mRNAs in the “neuron differentiation” category (179 mRNAs up-regulated in LII and 134 up-regulated in LDeep). We found that 116 differentially expressed mRNAs were also predicted targets of differentially expressed miRNAs. 11 of these mRNAs were transcription factors displaying negatively correlated expression patterns to the miRNAs predicted to target them, and seven of these eleven transcription factors contained highly conserved target sites with context scores below −0.1 (Fig. 2d, e). Some of these differentially expressed mRNAs, i.e., Sox5, Gli3, and Lmo4, are known to be drivers of laminar subtype specification in other brain areas (Ohtaka-Maruyama and Okado 2015; Woodworth et al. 2012), making these mRNAs and the miRNAs that regulate their expression prime candidates for transcriptional drivers of the laminar differences in neuron properties seen in the MEC.

### miRNA co-expression modules

**Fig. 3:**
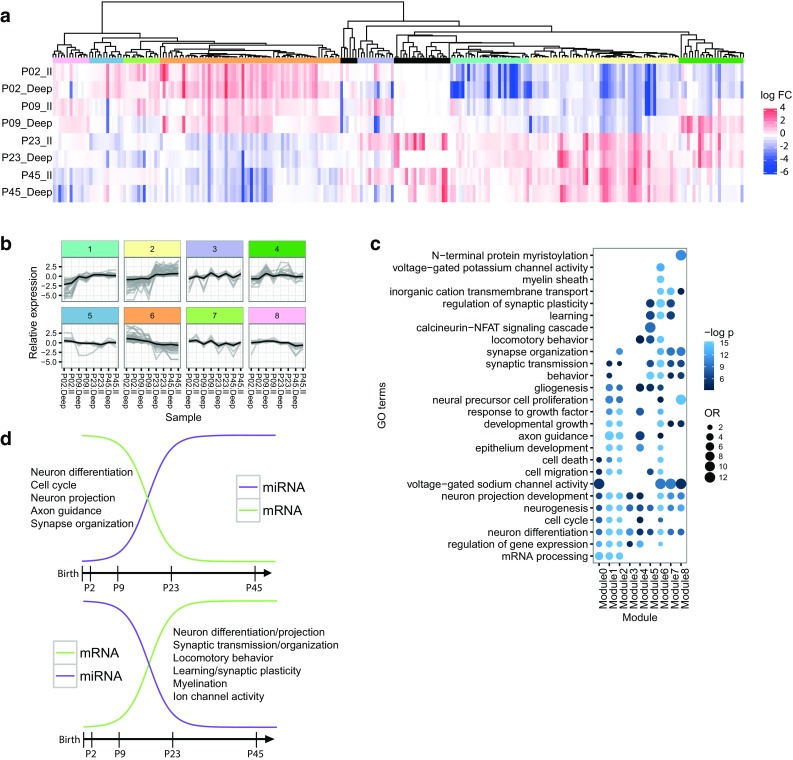
MicroRNA co-expression modules. **a** Differentially expressed miRNAs clustered according to Pearson correlation in a cluster dendrogram. The tree was cut using the recursive partitioning algorithm (Nilsen et al. 2013), yielding eight co-expression modules, each represented with its own color. Outliers are shown in *black*. **b** Relative expression of the miRNAs in the eight co-expression modules across the samples (*gray*) and the representative expression pattern of each module (*black*). Modules are *color* coded as in** a**.** c** Functional analysis of negatively correlated validated and conserved predicted target genes of the miRNAs in each co-expression module. The color intensity reflects the statistical significance (negative log adjusted *p* value), and the *size of the circles* the odds ratio calculated by Fisher’s exact test. For illustration purposes, all OR and negative log *p* values above a maximum value of 12 and 15, respectively, were rounded down to these maximum values. **d** Overview of the main gene ontology enrichment findings for temporally differentially expressed miRNAs. These findings are based on the negatively correlated, predicted targets of miRNAs with increasing (*left*) or decreasing (*right*) expression from the two early (P2/P9) to the two late time points (P23/P45)

There is increasing evidence that co-expressed miRNAs regulate functionally related genes (Bryan et al. 2014; Chavali et al. 2013; Gennarino et al. 2012; Guo et al. 2016; Wang et al. 2011). As our analyses of the miRNA expression data indicated multiple patterns of expression changes between MEC layers and postnatal ages (Fig. 1c–f), we clustered all miRNAs that were significantly differentially expressed (BH < 0.05) between ages or between layers. This approach identified eight robust co-expression modules that were representative of the 245 differentially expressed miRNAs (Fig. 3a, b, Supplementary Table 4). Consistent with previous observations (Baskerville and Bartel 2005) and with these modules representing co-regulated miRNAs, miRNAs encoded close in the genome tended to belong to the same co-expression module (Supplementary Analyses SA2, Supplementary Fig. 3 a–c).

The predominant patterns (modules 2 and 6) displayed opposing trends across development. Module 2 miRNAs displayed a sharp increase and module 6 a sharp decrease between P9 and P23. Laminar differences in miRNA expression were particularly seen for modules 3 and 7, which also had opposing trends between young and old animals. P9 had the highest number of miRNAs with laminar differences, and two modules were enriched in miRNAs that showed differential expression primarily at this age (modules 1 and 4). The miRNAs in module 5 were down-regulated in LII at P23 and P45, which makes it likely that the genes they regulate are up-regulated in LII at these time points.

### Correlating gene expression with miRNA expression

As most miRNAs either destabilize or repress their targets, we expected a majority of miRNAs and mRNA targets to display negatively correlated expression profiles. Indeed, when comparing the correlation distribution of conserved predicted targets to the control distribution of all predicted targets (including non-conserved), we found a skew towards negatively correlated miRNA-target pairs (Supplementary Fig. 4a). This skew was even more enhanced if we used more stringent criteria, such as increasing the TargetScan threshold, only including the 25% most highly expressed miRNAs, or requiring that an increasing percentage of the predicted miRNA target sites in a given gene were of miRNAs from the same module (Supplementary Fig. 4b–e). Combining all three filters gave a strong shift towards negative correlations compared to all conserved predicted targets (*p* = 6e-24, Mann–Whitney *U* test; Supplementary Fig. 4f). Although some miRNAs are known to increase the translation of their targets (Fabian et al. 2010), we chose to focus on these negatively correlated, conserved miRNA-gene pairs that are more likely to be real miRNA targets.

For the miRNAs in each of the co-expression modules, we identified validated targets from mirTarBase and predicted, conserved targets from TargetScan whose mRNA expression pattern was negatively correlated with that of the miRNA targeting it. The proportion of validated or predicted targets that were negatively correlated to the genes in each module varied from 7 to 50% for the different modules (Supplementary Table 4). Although we cannot exclude that this variation is an artifact of the analyses, we note that the miRNA module with the highest percentage of negatively correlated target genes had a clear developmental expression pattern, consistent with the importance of miRNA regulation of developmental genes (Ambros 2011; Davis et al. 2015).

### Functional analysis of the negatively correlated targets of miRNA co-expression modules

Based on the assumption that co-regulated miRNAs will target functionally related genes, we hypothesized that there would be a tendency for the target genes of each co-expression module to be enriched in GO terms that differ from those of the other modules. The functional enrichment analysis for the negatively correlated targets in each module (Fig. 3c) showed several general terms, especially those pertaining to general nervous system development, which were enriched across the modules. Modules 1 and 2 shared many enriched terms, which probably reflected their similarities in expression pattern. Most of their terms are linked to events that occur early in nervous system development, such as axon guidance and cell cycle and migration, indicating that the genes regulated by these miRNAs are down-regulated later in development.

Modules 5–8 also shared similarities in both expression patterns and enriched terms, including synaptic transmission, plasticity and locomotory behavior terms. Module 6 miRNAs may also be involved in regulating myelination; indeed, the expression patterns of module 6 miRNAs correspond with the onset of myelination from P10. The pattern of module 6 also corresponds with the maturation of grid cell properties from eye opening until P22 in the MEC. Modules 6–8 are enriched in ion transmembrane transport terms, implying that miRNAs could contribute to the maturation of physiological properties around the third postnatal week. Module 7, which has a clear laminar profile of miRNAs up-regulated in LII, may contribute to the differences in neuronal populations in the MEC layers. Predicted target genes include Hcn1, Scn1a/2a/8a, and Scn4b, but the negative correlation of miRNAs in module 7 with these ion channels seem to be more linked to developmental than laminar differences, as their expression increases across development. Hcn1 is important for grid cell function (Giocomo et al. 2011), and is likely targeted by miR-16 in module 7. In comparison, module 3 shared laminar profile with module 7 but had opposite expression across development. The two modules shared terms related to development and differentiation, but terms related to neuronal function such as sodium channel activity and synaptic transmission, were exclusive to module 7.

Finally, module 4 consisted of miRNAs that mainly were up-regulated in LDeep at P9. Although this module shared functions with other modules (primarily modules 1, 2, and 6), the strong laminar difference at P9 could indicate that the miRNAs in this module may contribute to initiate laminar differences in these functions, such as locomotory behavior, around this age.

In summary, the two predominant patterns (modules 2 and 6), shared functions such as growth, development, and cell migration, but also had distinct terms (Fig. 3d). MicroRNAs up-regulated at P23 and P45 (module 2) were enriched for mRNA processing and had markedly lower *p* values for cell cycle and axon guidance functions. In contrast, miRNAs down-regulated at P23 and P45 (module 6) were enriched for terms related to myelination, ion channel activity, synaptic plasticity, and locomotory behavior. Several of these terms were shared with modules that had similar but less pronounced expression differences between early and late ages (modules 5, 7, and 8). Our results implicate miRNAs in the development of the MEC’s navigational functions, as well as the specialized functions of neuronal subpopulations in the different layers.

### miRNA expression in retrogradely labeled stellate cells

**Fig. 4:**
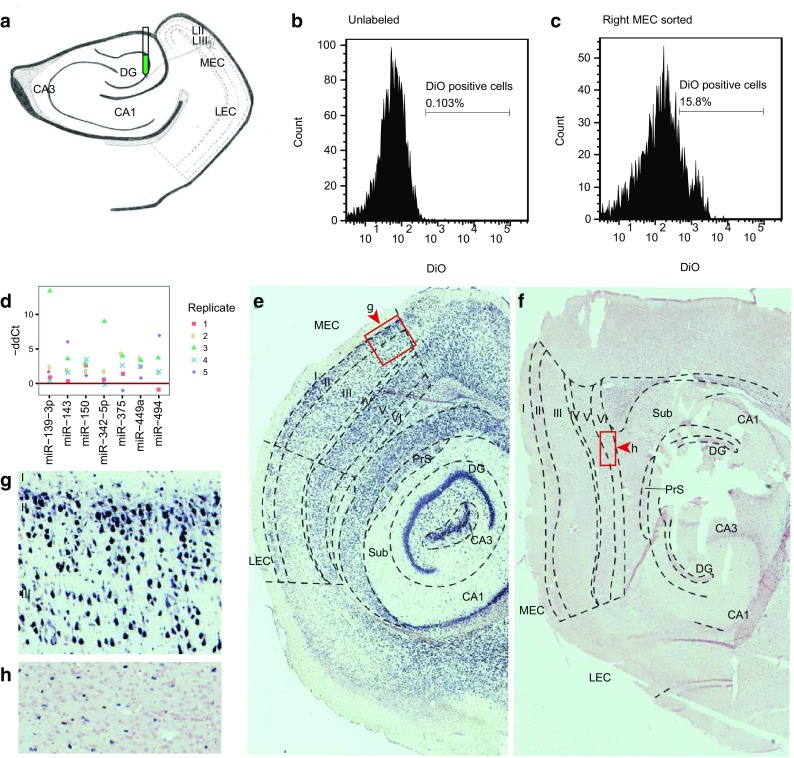
Differentially expressed miRNAs between FACS sorted retrogradely labeled stellate cells and non-labeled MEC cells, and validation of differentially expressed miRNAs by in situ hybridization. **a** Illustration of the retrograde labeling of the stellate neurons. DiO was injected by iontophoresis into the dentate gyrus, where the stellate neurons project. **b** DiO baseline fluorescence level in live dissociated, unlabeled MEC. This control was used to determine the fluorescence level threshold for the sort. **c** A fluorescence plot of a representative sample of live DiO labeled, dissociated MEC cells. The threshold for the fluorescence level used for the sort is shown. **d** miRNAs differentially expressed between stellate neurons and the rest of MEC. The five biological replicates are color coded and represented with different symbols.** e**–**h** miRNA *in situ* hybridization on sagittal brain slices from a P23 rat using LNA-probes for miR-143 (**e, g**) and miR-219-5p (**f, h**). Zoomed in areas (**g, h**) correspond to the labeled *boxed areas* in **e** and **f**, with *arrowheads* pointing to the *top* in **g** and **h**. The probe stain is *purple*, and the counter *stain red*

Although the stellate cells are the dominant cell type in MEC LII, it is possible that miRNAs could be up-regulated in LII without having stellate-specific expression. To identify miRNAs that are differentially expressed in stellate cells vs. the surrounding neurons and non-neuronal tissue, we retrogradely labeled stellate cells through injection of the fluorescent dye DiO into the dentate gyrus, which is a main site of stellate cells’ axonal projections (Tamamaki and Nojyo 1993) (Fig. 4a). The labeled cells were separated by FACS from the remaining tissue after tissue dissociation of dissected entorhinal cortex from pups aged P4/P5 (Fig. 4b, c, Supplementary Fig. 5). The young age of the pups allowed for fast diffusion of the dye and easy cell dissociation of young, unmyelinated neurons (Brewer and Torricelli 2007).

After miRNA expression analysis of each cell population by TaqMan miRNA qPCR array, seven miRNAs were found to be up-regulated in the labeled cells, while zero miRNAs were down-regulated (Fig. 4d, see “Methods”). When comparing these findings to those of the laminar sample study, we saw that one of the up-regulated miRNAs, miR-143, was also up-regulated in LII. Another miRNA, miR-150, was significantly up-regulated in LII at P09, and showed the same trend at P23 and P45, although up-regulation did not reach significance at these ages. Two of the other miRNAs, miR-375 and miR-494, also showed the same pattern of up-regulation in LII without reaching statistical significance in the layered samples. In general, we observed good correlation between the different technologies used (Spearman’s rank correlation 0.61–0.70, *p* < 3.5e-14; Supplementary Analyses SA3, Supplementary Fig. 6). Taken together, these results confirm that several of the miRNAs up-regulated in LII also are up-regulated in stellate cells and identify miR-143 as the prime candidate for a stellate-enriched miRNA.

### In situ hybridization of miRNAs

Although stellate cells were profiled at an early postnatal time point (P4/5), the results from the laminar samples showed that the expression of miR-143 increased further in LII across development. Due to this increased expression during postnatal development, it is possible that the importance of gene regulation by miR-143 increases with age. Grid cells, which are presumed to be stellate cells, reach maturation around the third postnatal week in rats, making the P23 developmental time point relevant for validation of miR-143 up-regulation. We hypothesized that if miR-143 was important for regulating stellate-specific gene expression, we would observe miR-143 expression in stellate cells of MEC. We therefore performed in situ hybridization using sagittal slices from brains of P23 rats.

The miR-143 signal was indeed strong in LII, with the signal in stellate neurons being very dense but not exclusive to this neuronal subtype (Fig. 4e, g). We noticed additional staining in smaller cells in LII, likely representing pyramidal principle cells and interneurons, as well as pyramidal cells in LIII and LV. miR-143 is known to be involved in differentiation and proliferation of vascular smooth muscle cells (Rangrez et al. 2011), and modulates the angiogenic and vessel stabilization properties of endothelial cells (Climent et al. 2015). Although we did see some staining in vessels, this signal was not universal across all vascular cells. Consequently, the vascular role of miR-143 appears to be less important in rat MEC, where it instead appears to have roles in stellate and pyramidal cell function. Interestingly, the density of miR-143 seemed to be higher in MEC than in the lateral part of the EC (LEC), and the signal was more homogeneous across layers in LEC. The medial and lateral entorhinal cortices are known to differ in electrophysiology, connectivity, and function, and with differing patterns in the two regions, miR-143 could be involved in regulating these properties.

We also examined miR-219-5p, which was the most significantly up-regulated miRNA of the deeper layers compared to LII, and which was not detected in the FACS-sorted stellate cells. At P2/P9, miR-219-5p had similarly low expression in LII and LDeep compared to the later time points, but its expression increased between P9 and P23, reaching a maximum level at P23/P45 when it also had a distinct laminar profile (Fig. 1f). Examination of this miRNA allowed us to further characterize the cell-specific localization of its expression and thus its role in MEC lamination. Consistent with the microarray data, there was hardly any miR-219-5p signal in LII, a weak signal in LIII, and a much stronger signal in LV and VI (Fig. 4f). The miRNA was expressed in ependymal cells, oligodendrocytes and glia in the tissue, which corroborates the findings of others, who have detected expression of this miRNA in glia and found it to be involved in oligodendrocyte differentiation (Zhao et al. 2010).

### Functional analysis of predicted targets of miRNAs differentially expressed in stellate cells indicates that the miRNAs are involved in stellate cell specialization

**Fig. 5:**
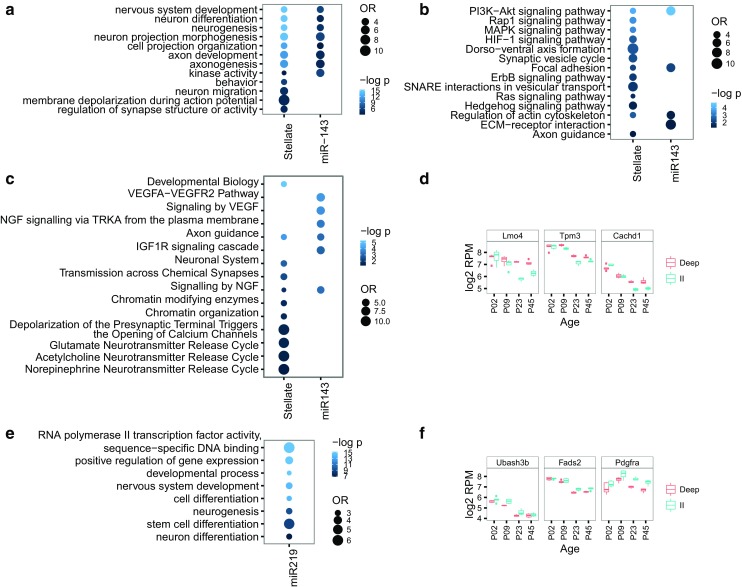
Analyses of predicted, conserved mRNA targets expressed in the MEC of miR-143 (**a–d**) and miR-219 (**e, f**). **a** Gene ontology, **b** KEGG pathway, and **c** REACTOME pathway enrichment analyses for validated and predicted, conserved targets of miRNAs up-regulated in stellate cells in general and miR-143 in particular. **e** Gene ontology enrichment analysis for validated and predicted, conserved targets of miR-219-5p. The color intensity reflects the statistical significance (negative log adjusted *p* value), and the *size of the circles* the odds ratio calculated by Fisher’s exact test. For illustration purposes, all OR and negative log *p* values above a maximum value of 12 and 15, respectively, were rounded down to these maximum values. Expression patterns of mRNAs that are the most likely miR-143 (**d**) and miR-219-5p (**f**) targets (rho < −0.5 and the best TargetScan 6.0 or 7.0 context score). Laminar samples are colored (LDeep *red*, LII *turquoise*)

The stellate cells were extracted from pups aged P4/P5, when there is marked synaptogenesis and neuron differentiation (Semple et al. 2013). Indeed, for the predicted or validated targets of the miRNAs up-regulated in labeled stellate cells, most of the significant terms were related to formation and differentiation of neurons (Fig. 5a). Enriched terms also included cell projection organization, behavior, and terms related to synaptic activity, whereas enriched pathways included PI3K-Akt, MAPK, and NGF signaling (Fig. 5b, c).

Because miR-143 was up-regulated both in stellate cells and in LII in general, we also specifically considered the enriched terms and pathways of its predicted or validated targets (Fig. 5a–c). As this miRNA also was included in the analysis of all the up-regulated stellate miRNAs, many of the same terms were enriched. However, what differed from the larger analysis was IGF1R activity and assembly of collagens. The IGF signaling pathway has a role in dendrite formation and synaptogenesis (Popken et al. 2005). Although the most characterized role for miR-143 is in vascularization, this miRNA could, by regulating IGF signaling, also have a special role in neurons.

### Correlating gene expression with miR-143 expression

To further delineate targets relevant for miR-143 in MEC LII and stellate cells, we combined the expression profile of miR-143 with those of its predicted, conserved targets. We required predicted targets to have negatively correlated expression patterns, a minimum expression level (median normalized log_2_ expression ≥ 5), and be significantly differentially expressed both between ages and between layers. Twelve genes satisfied these requirements (Supplementary Table 5). The most likely targets of miR-143 in the MEC, according to these criteria, were the Lmo4, Tpm3, and Cachd1 genes (Fig. 5d). Lmo4 was the gene with highest LFC between MEC layers in gene expression, the best target site as measured by the TargetScan context score, and the second highest negative correlation with miR-143 expression levels.

### Correlating gene expression with miR-219-5p expression

Predicted targets of miR-219-5p, irrespective of correlation with miR-219-5p expression, were enriched in neuron development terms (Fig. 5e). However, known targets of miR-219-5p include several genes involved in the process of differentiating neuronal stem cells to myelinating oligodendrocytes (Barca-Mayo and Lu 2012). All of the known targets that are involved in the development of oligodendrocytes from oligodendrocyte precursor cells displayed negatively correlated expression patterns with miR-219-5p, although few were differentially expressed between layers (Supplementary Analyses SA4, Supplementary Fig. 7). However, the gene involved in final maturation and myelin maintenance, Elovl7, had a positively correlated expression pattern with miR-219-5p.

Using the same criteria as for miR-143, we found 16 genes that had opposite expression to miR-219-5p between both ages and layers. Of these, we identified three genes that had both high context scores in TargetScan (v. 6.2 and 7.1) and a high degree of negative correlation, namely Fads2, Pdgfra, and Ubash3b (Supplementary Table 5; Fig. 5f).

## Discussion

### Known functions of MEC miRNAs differentially expressed between early and late postnatal ages or between layer II and deep layers

We found 202 miRNAs to be significantly differentially expressed between early (P2/P9) and late (P23/P45) ages or between LII and LDeep at individual time points. Most (192) of these were differentially expressed between ages (Fig. 1c; Supplementary Table 2). Three of the top five most significant miRNAs that increased from early to late age were members of the miR-29 family. MicroRNA miR-29b is known to increase during neuronal maturation and to inhibit apoptosis in neurons, and miR-29a/b both affect dendritic spine morphology (Kole et al. 2011; Lippi et al. 2011). Other general functions for this family include regulation of the extracellular matrix and cell proliferation and differentiation (Kriegel et al. 2012). miR-29a/b also target several proteins involved in neurodegenerative diseases, including BACE1/β-secretase, of which elevated levels can lead to increased amyloid β-peptides in patients with sporadic Alzheimer’s disease (Hebert et al. 2008). One of the miRNAs displaying the greatest decrease in expression between ages, miR-298, also targets the BACE1 mRNA (Boissonneault et al. 2009), suggesting complementary roles between miR-298 and miR-29a/b in regulating this protein. The other highly significant miRNAs displaying a steep decrease from younger to older animals (miR-301b, miR-130b, miR-20a, and miR-15b) are known to be involved in cancers (Attar et al. 2012; Funamizu et al. 2014; O’Donnell et al. 2005; Zhu et al. 2015), suggesting a role for these miRNAs in regulating MEC cell proliferation immediately after birth.

Only 35 miRNAs were significantly differentially expressed between LII and LDeep at individual ages (Fig. 1e; Supplementary Table 2); of these, 25 miRNAs differed significantly between early (P2/P9) and late (P23/P45) ages. The trend of a higher number of miRNAs being differentially expressed across development than between cortical layers, has also been observed in mouse somatosensory cortex (Fertuzinhos et al. 2014), and probably reflects the importance of miRNAs in the processes important for brain development in general. Between P2 and P23, there is extensive spine formation, synaptic pruning and myelination in the brain, all known to be regulated by miRNAs (Schratt 2009).

The two miRNAs with the lowest *p* value up-regulated in LII (miR-143 and miR-126; Fig. 1f) are involved in angiogenesis (Climent et al. 2015; Sonntag et al. 2012). Uneven laminar distribution of capillaries has been observed in the lateral part of the EC (Michaloudi et al. 2005), and it is possible that miRNAs could contribute to the formation and maintenance of a similar capillary pattern in the MEC. The two miRNAs with the lowest *p* value up-regulated in LDeep (miR-219 and miR-338) are involved in oligodendrocyte differentiation (Barca-Mayo and Lu 2012). The increased need for oligodendrocyte regulation in the deeper layer can be expected because there are higher levels of myelination in deeper cortical layers (Lodato et al. 2015). Several of the differentially expressed miRNAs regulate neuron differentiation, including miR-126 and miR-26b in LII, and miR-7a/b in LDeep. Interestingly, the LII up-regulated miR-26b and miR-126 are implicated in Alzheimer’s disease (Absalon et al. 2013; Kim et al. 2014), while the LDeep up-regulated miR-219 and miR-7a/b are implicated in schizophrenia (Beveridge and Cairns 2012), diseases which both show pathologies in LII.

In summary, the known functions of the miRNAs we identified as differentially expressed in MEC, indicate that these miRNAs regulate laminar differences in MEC vascular structure and oligodendrocyte density and could contribute to laminar differences in neuron subtype specification and disease susceptibility.

### Possible functions of MEC miRNA co-expression modules

By clustering the differentially expressed miRNAs, we found eight distinct patterns of co-expressed miRNAs. As expected from the statistical results (Fig. 1c), the two largest such co-expression modules had opposing patterns and contained miRNAs that primarily were differentially expressed between the early (P2 and P9) and late (P23 and P45) ages (modules 2 and 6 with 53 and 64 miRNAs, respectively; Fig. 3b; Supplementary Table 4). This probably reflects the enormous strides in development that take place between and around ages P9-P23, starting with crawling and eye opening, and ending with grid cell stabilization and the development of spatial learning abilities (Langston et al. 2010; Wills and Cacucci 2014).

To determine potential functions of the co-expressed miRNAs, we identified predicted and verified target mRNAs with negatively correlated expression to all miRNAs in each module and ran GO analyses of each set of such predicted targets. This approach relies on the assumptions that co-expressed miRNAs regulate functionally related genes and that filtering predicted miRNA targets based on negative correlation will reduce false positive predictions and identify physiologically relevant targets. Supporting the first assumption, miRNAs clustered in the genome, which tend to be co-transcribed and therefore co-expressed, tend to target functionally related genes (Hausser and Zavolan 2014; Wang et al. 2016). Similarly, other co-expressed miRNAs also tend to target functionally related genes (Bryan et al. 2014).

As for using negative correlations in expression to identify relevant miRNA targets, multiple studies have shown that predicted miRNA targets tend to be down-regulated upon miRNA overexpression, and vice versa upon miRNA down-regulation [reviewed in (Bartel 2009)]. Moreover, these changes in target mRNA expression generally result in the corresponding changes in protein levels (Baek et al. 2008; Selbach et al. 2008); indeed, miRNA’s effect on mRNA expression can explain most (>80%) of the changes in protein levels for predicted targets (Guo et al. 2010). Similar results have been seen for experimentally validated miRNA targets (Hendrickson et al. 2009). These studies show that experimentally altering miRNA expression generally results in inverse expression changes for predicted miRNA targets, but miRNA and predicted targets also tend to be negatively correlated in endogenous expression data (Fulci et al. 2009; Wang and Li 2009). Consistent with these previous studies, we found that the correlations between MEC miRNAs and their evolutionary conserved predicted miRNA targets are skewed towards negative values (Supplementary Fig. 4a).

Our GO analyses found that the miRNA co-expression modules had several functions in common. All modules were significantly enriched for predicted targets involved in neuron differentiation and neurogenesis (Fig. 3c). The two largest modules, representing miRNAs differentially expressed between early and late ages, shared several predicted functions, including growth, development, and cell migration. Although many of the significant GO terms appear general, our results indicate distinctions in the predicted functions of miRNAs up-regulated at P2/9 compared with miRNAs up-regulated at P23/45 (Fig. 3d). MicroRNAs up-regulated at P2/9, and thereby down-regulated at P23/45, predict that myelination, ion channel activity, learning, and synaptic plasticity are up-regulated. In contrast, cell cycle functions are predicted to be down-regulated at these later time points. Together these data indicate that the miRNAs are involved in the development of the MEC. Importantly, the miRNAs appear to be involved in regulation of maturational processes which lead to specialized cell functions that require specific ion channels. Furthermore, these data are in line with the idea that learning and synaptic plasticity may be more prominent at older ages, and indicate that miRNAs play a role in these functions.

### Possible functions of miRNAs up-regulated in stellate cells

GO analyses of predicted and validated targets of miRNAs up-regulated in the FACS-sorted stellate cells identified functions related to neuronal development and differentiation (Fig. 5a). Although neurogenesis in the EC largely takes place during embryonic development (Bayer 1980), “neurogenesis” as a GO term could reflect the reuse of similar pathways in processes that are known to take place postnatally, such as morphological and functional maturation (Casanova and Casanova 2014), or that the same miRNAs target genes involved in neurogenesis during embryonic development and other genes later in development. Many of the enriched terms, such as neurogenesis, neuron differentiation, and axonogenesis, were also among the terms enriched for mRNA and miRNAs up-regulated in LII.

Enriched terms for the stellate miRNAs also included more specific functions such as “regulation of synapse structure or activity” and “membrane depolarization during action potential”. Pathways enriched for the stellate miRNAs included NGF, MAPK, and PI3K-Akt, which contribute to a wide variety of both intra-and extracellular processes, including differentiation of neurons (Berry et al. 2012; Correa and Eales 2012; Peltier et al. 2007). Fine-tuning of these pathways by miRNAs could potentially contribute to the stellate phenotype.

### Likely targets of miR-143 and miR-219-5p

The top predicted target genes of miR-143, Lmo4, Tpm3, and Cachd1, indicate that miR-143 contributes to laminar and subcellular phenotypes of MEC. The Lmo4 gene, whose expression is enriched in glutamatergic populations, is an activity-dependent calcium-responsive cofactor that binds to several signaling molecules and transcription factors (Qin et al. 2012). Lmo4 is involved in the establishment of neuronal subtypes in the rostral motor cortex and in LV cortical neuron populations (Azim et al. 2009; Cederquist et al. 2013) and is implicated in hippocampus-dependent spatial learning (Qin et al. 2012). In addition, Lmo4 is strongly expressed in MEC during embryonic development, and has been implicated in characterizing this region (Abellan et al. 2014). It is therefore possible that the lower concentration of Lmo4 gene expression in LII is important for the specialization of LII. The Tpm3 protein is a component of actin microfilaments, and certain isoforms have been implicated in influencing the size and shapes of neurons (Schevzov et al. 2005). The function of Cachd1 has not been investigated, but it may be a calcium channel regulatory membrane protein as inferred from gene ontology inferred electronic annotation (Gene Ontology Consortium).

The top predicted gene targets of miR-219-5p, Fads2, Pdgfra, and Ubash3b, indicate a role for this miRNA in regulation of myelination. The Pdgfra gene is a known target of miR-219-5p involved in differentiation of oligodendrocyte precursor cells (OPCs) into mature oligodendrocytes (Barca-Mayo and Lu 2012), which corresponds well with the *in situ* labeling of miR-219-5p in oligodendrocytes presented here. Oligodendrocytes are the main cell types involved in myelination, and the Fads2 gene, a second target of miR-219-5p, is also involved in myelination (Peters et al. 2014). Further support for the role of miR-219-5p in myelination is that Ubash3b (alias Sts-1) inhibits endocytosis of EGFR (Raguz et al. 2007) and therefore possibly plays an indirect role in oligodendrocyte development, which involves EGFR signaling (Palazuelos et al. 2014). Thus, miR-219-5p likely regulates oligodendrocyte differentiation in the MEC in a layer-specific fashion, a role which is similar to its known function in other brain areas.

## Conclusions

We have presented the first analysis of miRNA expression in LII and LDeep of the developing MEC, with a special focus on stellate neurons. We have profiled miRNAs and mRNAs in MEC LII and LDeep at four time points during postnatal development—neonatal (P2), infant (P9), juvenile (P23) and young adult (P45)—and compared miRNA expression in labeled stellate cells to that in other MEC cells. Through in situ hybridizations, we confirmed the layer and cell-type expression of miR-143, which is up-regulated in LII stellate cells but also expressed in smaller cells in LII and LIII and LV pyramidal cells, and miR-219-5p, which is expressed in ependymal cells, oligodendrocytes, and glia—primarily in LV and LVI. Our analysis showed that both miRNAs and mRNAs were more dynamic across development than they were between layers. However, we did find laminar differences in miRNA expression at all postnatal ages tested. Moreover, our bioinformatics analyses of conserved, predicted target mRNAs with negatively correlated expression patterns indicate that these miRNAs could participate in regulating the laminar differences in electrophysiology, neuron morphology and disease susceptibility seen in the MEC.

The miRNA most significantly up-regulated in LDeep, miR-219-5p, plays an important role in oligodendrocyte differentiation and myelination, which generally begins around P10. The most significantly up-regulated miRNA in LII, miR-143, was found to be up-regulated particularly in stellate neurons. Our analysis revealed that a likely target of this miRNA is the Lmo4 gene, which is important for neuronal subtype specification and hippocampus-dependent spatial learning. The exact role of miR-143 in Lmo4 gene regulation and its potential importance for stellate or grid cell function remains to be determined.

## Electronic supplementary material

Below is the link to the electronic supplementary material.

Supplementary material 1 (DOCX 543 KB)

Supplementary material 2 (XLSX 10 KB)

Supplementary material 3 (XLSX 29 KB)

Supplementary material 4 (XLSX 1248 KB)

Supplementary material 5 (XLSX 22 KB)

Supplementary material 6 (XLSX 18 KB)
