# Supplementary material for: MicroRNAs contribute to postnatal development of laminar differences and neuronal subtypes in the rat medial entorhinal cortex
Source: Brain Struct Funct. 2017 Mar 4;222(7):3107–26. doi: 10.1007/s00429-017-1389-z (PMC5585308; doi:10.1007/s00429-017-1389-z)
Supplement: Supplementary file 1 — Supplementary material 1 (DOCX 543 KB) [file 429_2017_1389_MOESM1_ESM.docx]

# MicroRNAs contribute to postnatal development of laminar differences and neuronal subtypes in the rat medial entorhinal cortex

Lene C. Olsen^1^, Kally C. O’Reilly^2^, Nina B. Liabakk^1^, Menno Witter^2^, and Pål Sætrom^1,3,4,*^

1. Department of Cancer Research and Molecular Medicine, Norwegian University of Science and Technology, Trondheim, Norway.
2. Kavli Institute for Systems Neuroscience and Centre for Neural Computation, Norwegian University for Science and Technology, Trondheim, Norway.
3. Department of Computer and Information Science, Norwegian University for Science and Technology, Trondheim, Norway.
4. Bioinformatics core facility - BioCore, Norwegian University of Science and Technology, Trondheim, Norway.

*)Correspondence and request for materials should be addressed to P.S. (e-mail: [pal.satrom@ntnu.no](mailto:pal.satrom@ntnu.no))

# Supplementary Materials


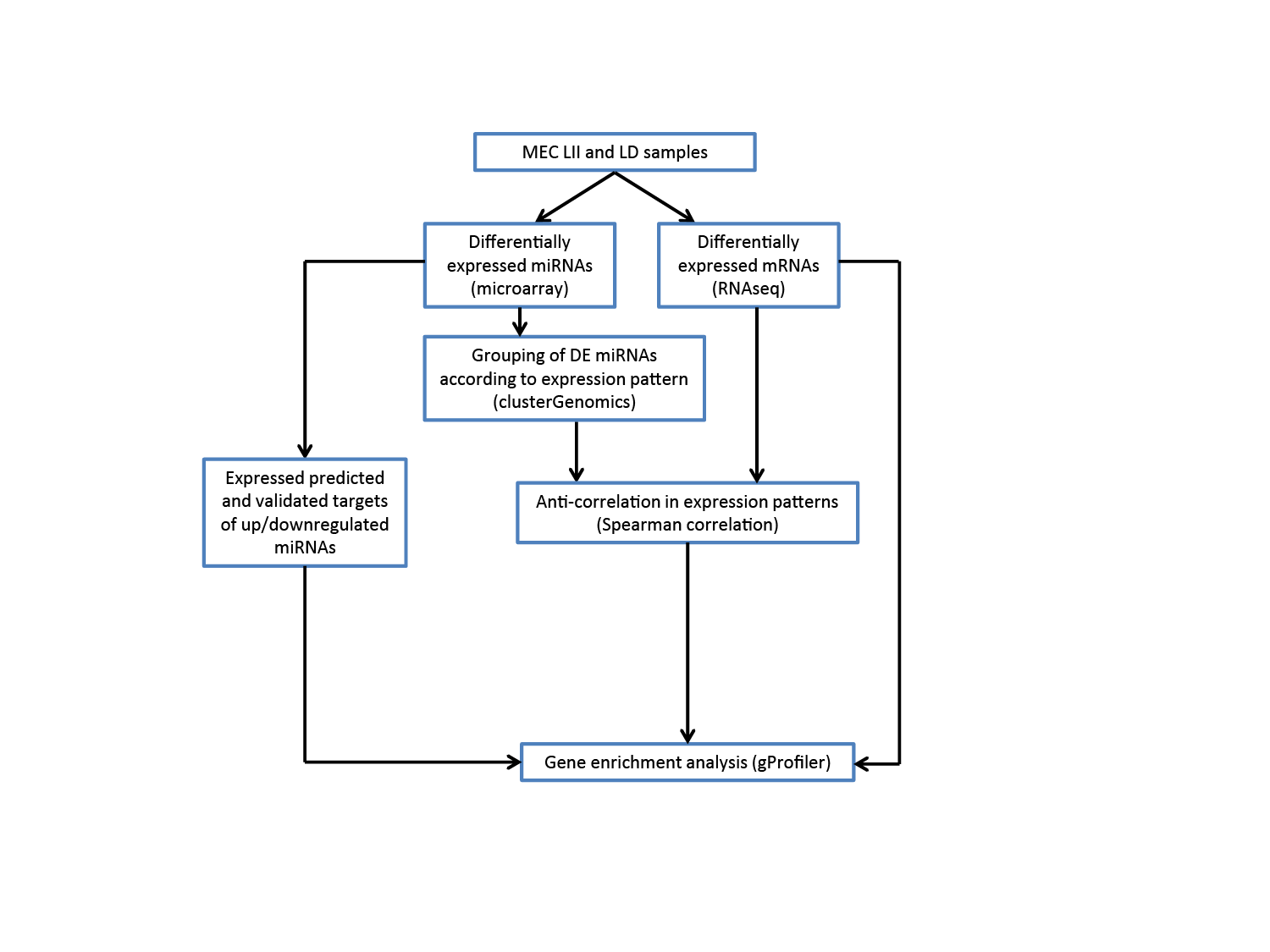


**Supplementary Fig. 1:** Summary of workflow for analysis of laminar samples. The samples were analyzed for differential miRNA and mRNA expression. Differentially expressed miRNAs were grouped according to correlating expression pattern, and the predicted target genes for the miRNAs in each group were analyzed for anti-correlative expression patterns. Anti-correlated targets, along with differentially expressed genes and all predicted and validated expressed targets of differentially expressed miRNAs were analyzed for enriched gene ontology terms.


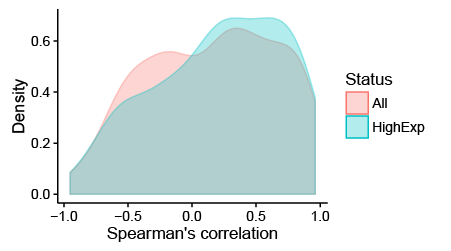


**Supplementary Fig. 2:** Correlation between the expression patterns across layers and development of the mature miRNAs as measured by microarray and the expression patterns of the pre-mirs as measured by deep sequencing. The correlation of all miRNA-pre-mirs in red, and of the miRNAs that are expressed with a minimum median normalized expression level of 5 (as determined by microarrays) in turquoise.


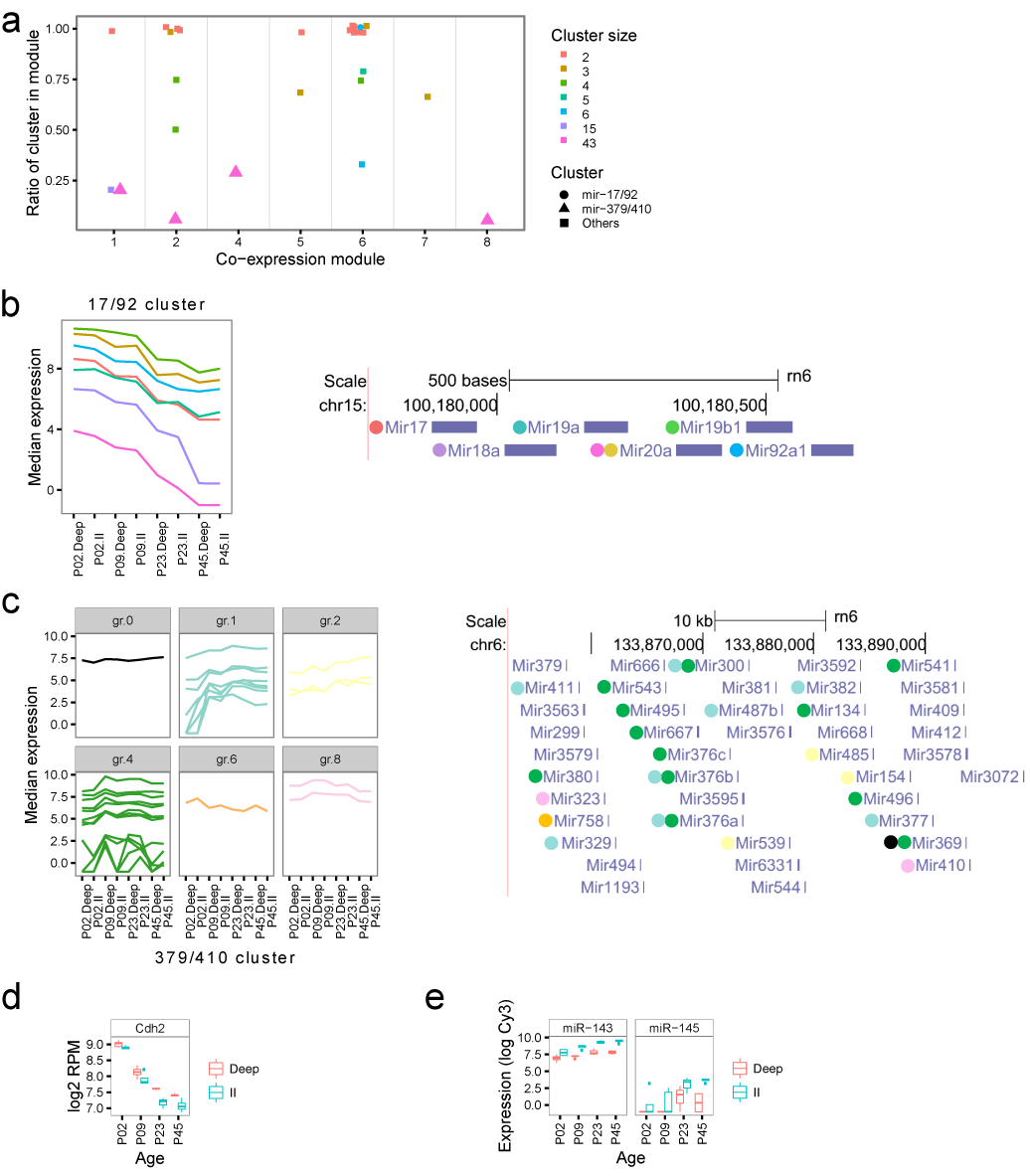


**Supplementary Fig. 3: a)** Co-expression patterns of miRNAs clustered in the genome. Each point represents miRNAs from a genomic miRNA cluster, grouped by their co-expression patterns and color coded by the number of miRNA hairpins in the genomic cluster. Module 3 is not presented because it did not contain any miRNA genomic clusters. The y-axis gives the proportion of miRNAs from the cluster that is present in the given module. All six members of the mir-17/92 cluster are present in module 6 (circle; see D), yielding a ratio of 1; members of the mir-379/410 cluster are present in four different modules (triangle; see E). **b)** Genomic organization of the members of the mir-17/92 cluster in the rat genome (rn6), along with median expression levels for each member of the cluster. The identity of each pre-mir is color coded. Pre-mir-20a is represented with two colors, with the yellow depicting the expression of the mature miR-20a and the magenta the mature miR-20a*. **c)** Genomic organization of the members of the mir-379/410 cluster in the rat genome (rn6), along with median expression levels for the members of the cluster present in each co-expression module. The color coding is according to co-expression module, gr.0 being the outlier group. **d)** The expression of the Cdh2 gene across time points. Laminar samples are colored as in Fig. 1. **e**) Comparison of expression patterns for miR-143 and miR-145, which belong to the same miRNA cluster.


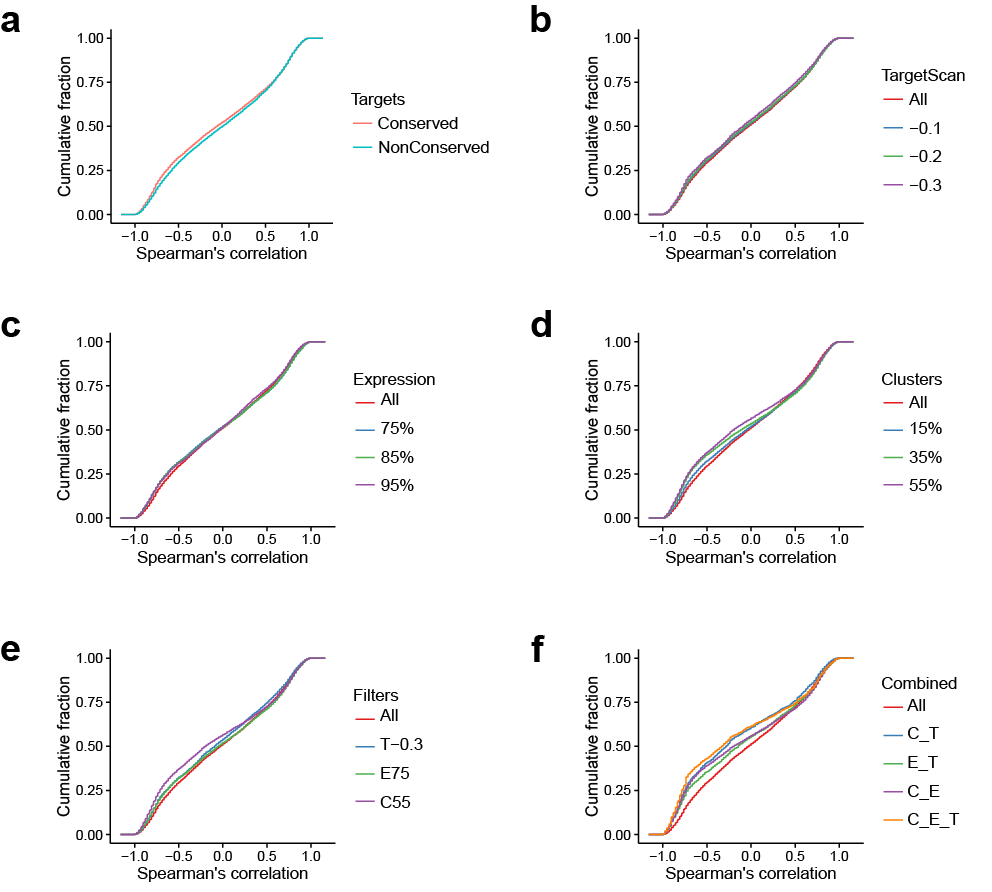


**Supplementary Fig. 4:** Cumulative distribution of the correlation between miRNA expression and the expression of predicted target genes. **a)** Curves show correlations for predicted conserved (red) and non-conserved (turquoise) target genes. The predicted conserved miRNA target genes are more skewed towards negative correlation values than those for non-conserved miRNA target genes. **b)** Curves show correlations for all predicted targets (red) and predicted targets with context scores of <-0.1 (blue), <-0.2 (green), and <-0.3 (purple). There is a shift towards more negative correlation when the context score threshold is decreased (corresponding to more stringent predictions). **c)** Curves show correlations for all predicted targets (red) and for the pairs where the miRNAs with the 75% (blue), 85% (green), and 95% (purple) lowest expression levels have been filtered out. The higher the expression of the miRNAs, the higher the tendency towards negative correlation. **d)** Curves show correlations for all predicted targets (red) compared to when at least 15% (blue), 35% (green), and 55% (purple) of the target sites in the predicted target genes are present in the same co-expression module. The higher the percentage of target sites targeted by miRNAs in the same co-expression module, the greater the shift towards negative correlation. **e)** Curves show correlations for all predicted targets (red) compared with the cumulative correlation when using the different kinds of filters (TargetScan context score < -0.3 in blue, miRNAs with expression levels above the 75^th^ percentile in green, and >55% of target sites covered by the same co-expression module in purple). **f)** Curves show correlations when using varying criteria for miRNA expression level, TargetScan context score, and coverage of predicted target sites by the miRNAs in the same co-expression module. All target gene – miRNA pairs (red) are compared to the pairs with the following combination of criteria: C_T (blue) are pairs where the target site has a context score <= -0.3 in TargetScan v. 6.0 and where >55% of the target sites are covered by the miRNAs in the same co-expression module (blue), pairs where the only miRNAs that have expression levels in the upper quartile are included (green), and pairs where the target site has a context score of <-0.3 in TargetScan v. 6.0 (purple). Pairs fulfilling all three criteria are shown in orange. All filters lead to a shift towards negative correlation, with the combination of all the filters displaying the highest degree of negative correlation.


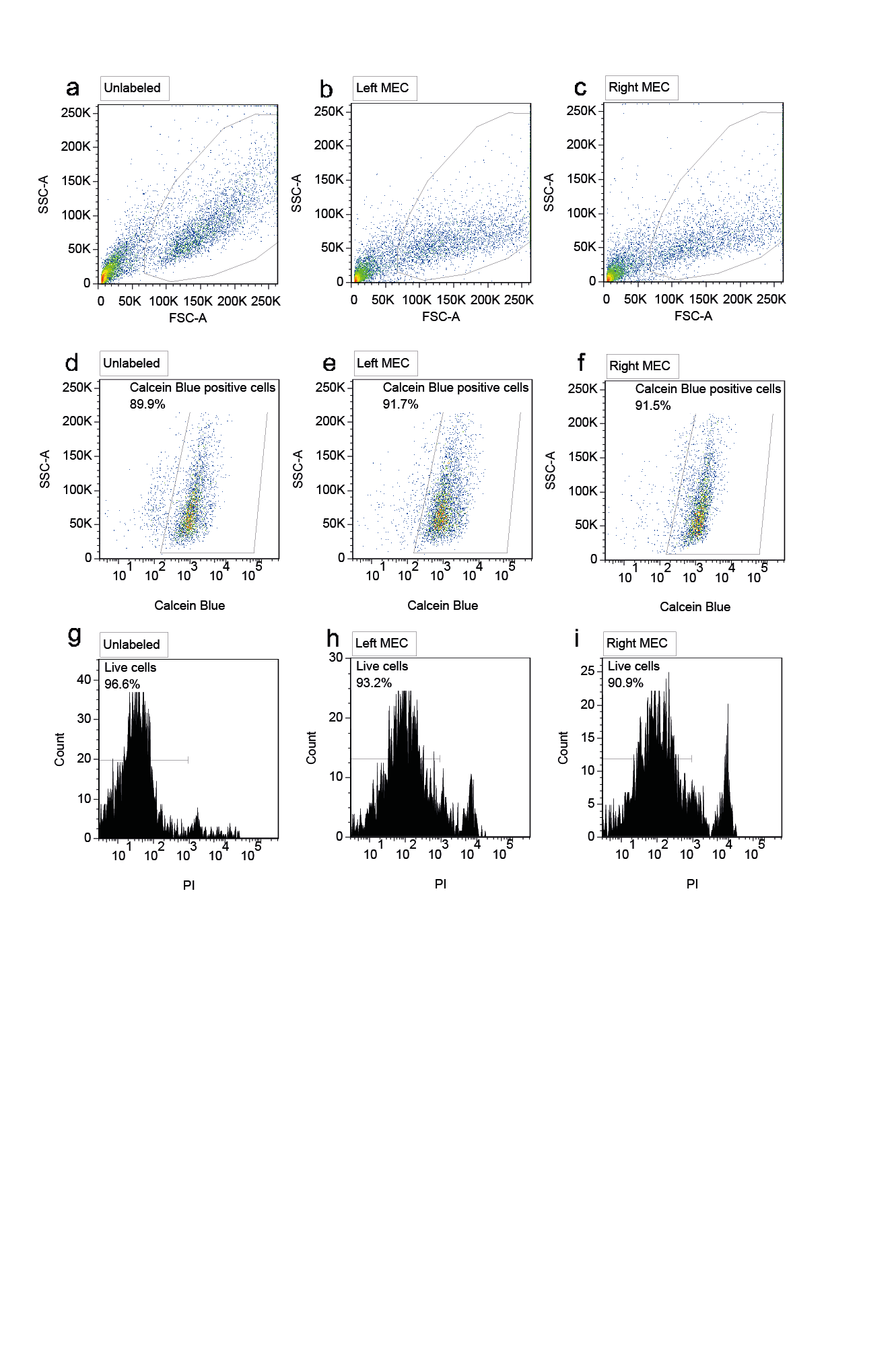


**Supplementary Fig. 5:** FACS gating. Forward and side scatter plot of a representative population of dissociated, unlabeled MEC showing gating of selected population for dissociated **a)** unlabeled MEC, **b)** labeled MEC from the left hemisphere, and **c)** labeled MEC from the right hemisphere. The gated population in A-C showed a high percentage of live cells as indicated by Calcein Blue staining in **d)** unlabeled MEC, **e)** labeled MEC from the left hemisphere, and **f)** labeled MEC from the right hemisphere. The bottom panel shows gating of PI negative cells for **g)** unlabeled MEC, **h)** labeled MEC from the left hemisphere, and **i)** labeled MEC from the right hemisphere. The FSC/SSC and PI gating were used for the sort.


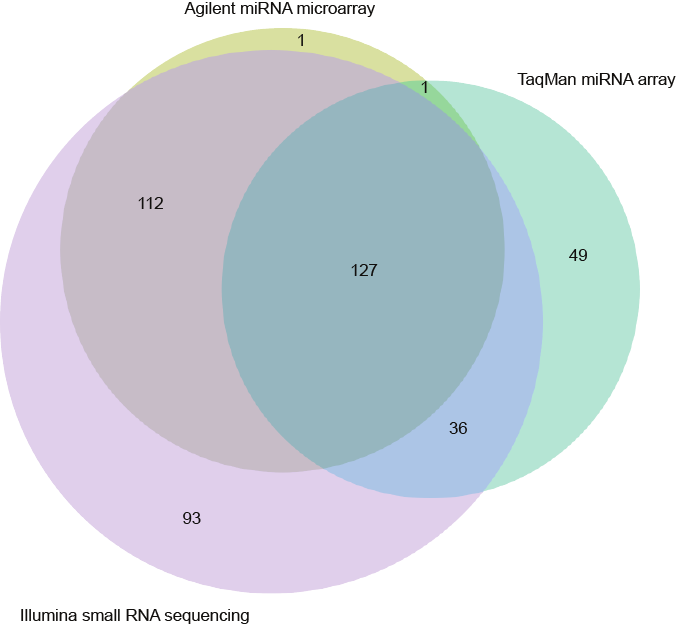


**Supplementary Fig. 6:** Venn diagram showing overlap of detected miRNAs in P23 MEC samples between the two array platforms used in this paper (Agilent miRNA microarray and TaqMan miRNA array) and Illumina small RNA sequencing of P23 MEC.


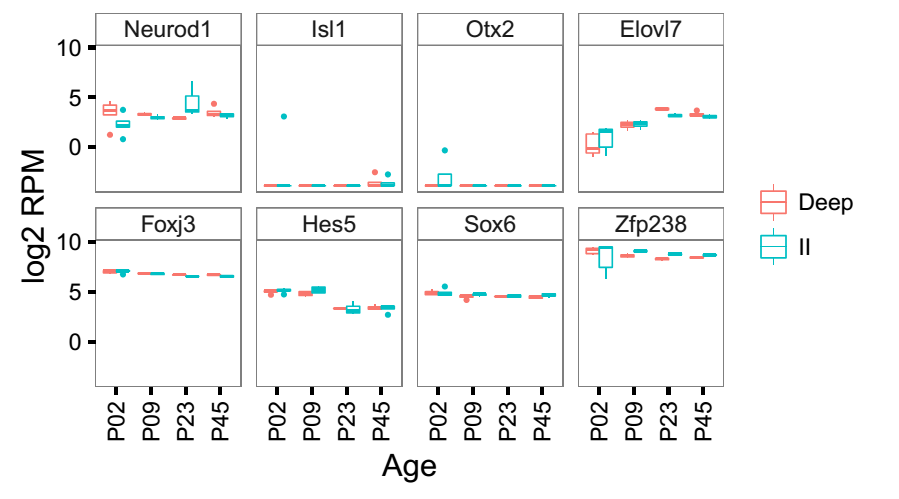


**Supplementary Figure 7:** Expression patterns of known miR-219-5p target genes important for oligodendrocyte differentiation.

# Supplementary Analyses

SA1: Correlation of precursor miRNA expression with mature miRNA expression

As the library preparation in our study included all RNA molecules, but with depletion of ribosomal RNAs, we were able to also identify a few precursor miRNAs (pre-mirs). Pre-mir-143 was upregulated in LII (LFC 1.17, BH p=0.01), providing further verification of its differential expression between MEC layers. The adjacent mir-145 was also detected by the RNA-seq, and showed the same trend in expression, but did not reach significance levels due to higher variation (Supplementary Fig. 3e). In general, the mature miRNA expression from the microarray experiment correlated positively with the pre-mir expression from the RNA-seq experiment (Supplementary Fig. 2). The shift was further pronounced when we only examined highly expressed miRNAs (mature miRNAs with median normalized expression level > 5). Thus overall, RNA-seq reads mapping to pre-mirs appear to be representative for miRNA expression in postnatal rat EC.

SA2: Genomically clustered miRNAs in co-expression modules

As miRNAs encoded close together in the genome (genomic clusters) tend to share expression profiles ([Thapa et al. 2015](#_ENREF_14)), we expected that such clustered miRNAs would be grouped in the same or similar co-expression modules. To test whether the grouping of miRNAs in our modules was consistent with known miRNA clusters, we examined the chromosomal locations of each miRNA within each module for clustering of miRNAs in the genome. Most modules contained at least one pair of genomically clustered miRNAs, except the outlier module and module 3 which contained no clustered miRNAs (Supplementary Fig. 3a).

The modules displaying the clearest developmental profiles (modules 2 and 6) contained several oncomiR and anti-oncomiR clusters, such as the miR-125a/let-7c/99b and miR-23b/27b/24-1 clusters. Module 6 contained all members of the miR-17/92 oncomiR cluster (Supplementary Fig. 3b) that is known to regulate the cell cycle and apoptosis, and has been implicated in neurodegenerative diseases ([Jovicic et al. 2013](#_ENREF_4); [Mogilyansky and Rigoutsos 2013](#_ENREF_8)). This cluster is also of particular importance for oligodendrocyte proliferation ([Petri et al. 2014](#_ENREF_10)). Thus, cell cycle, apoptosis, and oligodendrocyte proliferation is highly regulated by the miR-17/92 early in development, and this regulation decreases by P23 to adulthood.

Twelve of the 23 miRNAs in module 4 are members of the neuron-specific miR-379/410 cluster (Supplementary Fig. 3c), although other members of this large cluster of 42 miRNAs could also be found in module 1 (8 members), module 2 (3 members) and module 8 (2 members). There is little laminar difference for the miRNAs in the modules enriched in this cluster, apart from a downregulation in LII at P9 for some miRNAs. This cluster has been found to fine-tune the expression of N-cadherin ([Rago et al. 2014](#_ENREF_12)), thereby regulating neuronal differentiation and migration. N-cadherin (Cdh2) shows slight laminar differences (LFC -0.28, BH = 0.00016), but is strongly down-regulated in all layers from P9 (Supplementary Fig. 3d). The general expression pattern of the miR-379/410 cluster members is consistent with the majority of the miRNAs being up-regulated or having peak activity at P9 (modules 2 and 4), or being progressively turned on through development (module 2). The high expression at P9 is negatively correlated with the downregulation of the Cdh2 gene at the same age, which can indicate the regulation of this gene by the miR-379/410 cluster also in the MEC.

The miRNA with the most significant laminar difference, miR-143, was upregulated in LII. It is encoded in an intergenic region of the genome, close to miR-145. The expression of miR-145 followed a similar pattern to miR-143 (LFC=1.88, BH p=0.005), although at a much lower level (Supplementary Fig. 3e). According to miRBase, these miRNAs have similar expression levels in humans, but in mice miR-143 is expressed at a much higher level than miR-145, so this pattern seems common for rodents. These two miRNAs are commonly found at high levels in vascular smooth muscle cells in other tissues, where they play a role in angiogenesis and blood vessel stabilization ([Climent et al. 2015](#_ENREF_2)). The laminar expression difference for these miRNAs could therefore suggest laminar differences in MEC vascular structure, but could also indicate that these miRNAs have hitherto unrecognized brain-specific functions.

SA3: Comparison of miRNA detection between platforms

We used two different technologies for the miRNA expression studies, microarray for the layer samples, and TaqMan array for the stellate subtype samples. To investigate the technical issues of using different technologies, we compared detected miRNAs in the microarray layer experiment (P23 median miRNA expression across MEC layers) and the TaqMan array study (miRNA expression of total RNA from whole MEC P23) with Illumina small RNA sequencing data of total RNA from whole MEC at P23.

All of the three technologies have their own biases. Both Illumina sequencing and the TaqMan array use PCR, which can differ in amplification efficiency depending on the GC content of the cDNA ([Meyer and Liu 2014](#_ENREF_7); [Polz and Cavanaugh 1998](#_ENREF_11)). For the TaqMan array protocol we also used pre-amplification, which could potentially introduce more bias, although this technology has high sensitivity and accuracy for samples of low concentration ([Mestdagh et al. 2014](#_ENREF_6)). Another difference with the samples used for the TaqMan array is that they came from dissociated, FACS sorted cells. This process can be very stressful to the cells, which in turn could potentially alter the miRNA expression. However, results from Okaty *et al.* (2011) ([Okaty et al. 2011](#_ENREF_9)) showed no significant difference in the expression of stress, apoptosis, and immediate early genes between FACS and laser capture microdissection, indicating that FACS is not much more stressful for the cells than other technologies. As for the microarray technology, it has a more limited range than the other two technologies, and we could therefore have missed some differentially expressed miRNAs. Also, as with the TaqMan array, the microarray can only measure the concentration of miRNAs for which it has appropriate probes.

When comparing the platforms, we found 127 miRNAs to be detected by all three technologies (Supplementary Fig. 6). All but two of the miRNAs detected in the microarray experiment were detected with deep sequencing, of which one was also detected by TaqMan array. Four additional miRNAs were not detected in the deep sequencing analysis pipeline as they did not align to the genome (see Methods), but they were detected with reads in the raw data. Fourty-nine miRNAs were detected by the TaqMan array and not by deep sequencing or microarray. Only ten of these are found in v.21 of miRBase. The rest were putative miRNAs at the time of the design of the qPCR array, and are likely not real miRNAs. Five miRNAs detected by the TaqMan array did not align to the genome, but were detected in the raw reads.

The correlation between the different technologies was highest for small RNA sequencing and the TaqMan array (rho = -0.70, BH <2.2e-16), and lowest for the TaqMan array and microarray (rho = -0.61, BH 3.5e-14). This latter figure is higher than what Chen *et al.* ([Chen et al. 2009](#_ENREF_1)) found when comparing TaqMan array and microarrays. The correlation between microarray and small RNA sequencing was 0.64 (Spearman’s rho, BH <2.2e-16), which is slightly lower than what Tam *et al.* ([Tam et al. 2014](#_ENREF_13)) found when correlating Illumina small RNA sequencing and Illumina miRNA microarray (0.69). This slightly lower correlation could be due to differences in technologies, but could also be because of differences between the samples we used for sequencing and microarray analyses. The sample used for sequencing came from whole MEC, whereas the microarray samples were MEC layers from other rats than that used for sequencing. Despite these sample differences, correlations between technologies were similar to what has previously been found by others.

SA4: Analysis of known targets of miR-219-5p

miR-219-5p has several known targets involved in the differentiation of oligodendrocytes. Of the three target genes involved in early development from neural stem cells to OPCs (NeuroD1, Isl1, and Otx2), only NeuroD1 showed expression, and this was fairly stable across time (Supplementary Fig. 7). This result indicates that the OPC developmental stage is largely finished by the P2. The six known target genes involved in development from OPCs to myelinating oligodendrocytes (Elovl7, Foxj3, Hes5, Pdgfra, Sox6, Zfp238) were all expressed (Figure 5e, Supplementary Fig. 7), which was expected as myelination occurs during the age range tested (from P10, ([Downes and Mullins 2014](#_ENREF_3))). Expression of all of these genes, except for Elovl7, was negatively correlated with miR-219-5p expression, but only Pdgfra and Zfp238 displayed the opposite laminar pattern to the miRNA. The Hes5 gene, a powerful repressor of myelin gene expression ([Liu et al. 2006](#_ENREF_5)), showed a sharp downregulation in its expression between P9 and P23, which is when myelination begins.

# Supplementary References

Chen Y, Gelfond JA, McManus LM, Shireman PK (2009) Reproducibility of quantitative RT-PCR array in miRNA expression profiling and comparison with microarray analysis BMC Genomics 10:407 doi:10.1186/1471-2164-10-407

Climent M, Quintavalle M, Miragoli M, Chen J, Condorelli G, Elia L (2015) TGFbeta Triggers miR-143/145 Transfer From Smooth Muscle Cells to Endothelial Cells, Thereby Modulating Vessel Stabilization Circ Res 116:1753-1764 doi:10.1161/CIRCRESAHA.116.305178

Downes N, Mullins P (2014) The development of myelin in the brain of the juvenile rat Toxicol Pathol 42:913-922 doi:10.1177/0192623313503518

Jovicic A, Roshan R, Moisoi N, Pradervand S, Moser R, Pillai B, Luthi-Carter R (2013) Comprehensive expression analyses of neural cell-type-specific miRNAs identify new determinants of the specification and maintenance of neuronal phenotypes J Neurosci 33:5127-5137 doi:10.1523/JNEUROSCI.0600-12.2013

Liu A, Li J, Marin-Husstege M, Kageyama R, Fan Y, Gelinas C, Casaccia-Bonnefil P (2006) A molecular insight of Hes5-dependent inhibition of myelin gene expression: old partners and new players EMBO J 25:4833-4842 doi:10.1038/sj.emboj.7601352

Mestdagh P et al. (2014) Evaluation of quantitative miRNA expression platforms in the microRNA quality control (miRQC) study Nat Methods 11:809-815 doi:10.1038/nmeth.3014

Meyer CA, Liu XS (2014) Identifying and mitigating bias in next-generation sequencing methods for chromatin biology Nat Rev Genet 15:709-721 doi:10.1038/nrg3788

Mogilyansky E, Rigoutsos I (2013) The miR-17/92 cluster: a comprehensive update on its genomics, genetics, functions and increasingly important and numerous roles in health and disease Cell Death Differ 20:1603-1614 doi:10.1038/cdd.2013.125

Okaty BW, Sugino K, Nelson SB (2011) A quantitative comparison of cell-type-specific microarray gene expression profiling methods in the mouse brain PLoS One 6:e16493 doi:10.1371/journal.pone.0016493

Petri R, Malmevik J, Fasching L, Akerblom M, Jakobsson J (2014) miRNAs in brain development Exp Cell Res 321:84-89 doi:10.1016/j.yexcr.2013.09.022

Polz MF, Cavanaugh CM (1998) Bias in template-to-product ratios in multitemplate PCR Appl Environ Microbiol 64:3724-3730

Rago L, Beattie R, Taylor V, Winter J (2014) miR379-410 cluster miRNAs regulate neurogenesis and neuronal migration by fine-tuning N-cadherin EMBO J 33:906-920 doi:10.1002/embj.201386591

Tam S, de Borja R, Tsao MS, McPherson JD (2014) Robust global microRNA expression profiling using next-generation sequencing technologies Lab Invest 94:350-358 doi:10.1038/labinvest.2013.157

Thapa I, Fox HS, Bastola D (2015) Coexpression Network Analysis of miRNA-142 Overexpression in Neuronal Cells Biomed Res Int 2015:921517 doi:10.1155/2015/921517
